# Incidental Cardiac Computed Tomography Findings in Patients Undergoing Atrial Fibrillation Catheter Ablation

**DOI:** 10.7759/cureus.27886

**Published:** 2022-08-11

**Authors:** Mohamed Hamed, Martin Kloosterman, Eric Berkowitz, Jonathan Rosman, Joel Morris, Murray Rosenbaum

**Affiliations:** 1 Internal Medicine, Florida Atlantic University, Boca Raton, USA; 2 Cardiology, Florida Atlantic University, Boca Raton, USA; 3 Cardiac Radiology, Diagnostic Centers of America, Boca Raton, USA

**Keywords:** ct cardiac, collateral findings, catheter ablation, atrial fibrillation, cardiac computed tomography, incidental findings

## Abstract

Background: Catheter ablation (CA) is an effective technique for the management of atrial fibrillation (AF). Cardiac computed tomography (CCT) is a non-invasive imaging modality that is used as a crucial part of planning before CA procedures which can detect other incidental findings and require further diagnostic investigations.

Objectives: We sought to assess the prevalence and distribution of incidental CCT findings in patients with AF undergoing CA.

Methods: Retrospective analysis over a three-year period (2013-2016) of 218 patients undergoing CCT prior to AF CA. CCT findings were analyzed and incident clinically important findings were reported.

Results: Over the three-year period, 218 patients had undergone CCT. Of these, 28.8% showed clinically significant incidental findings in the chest and upper abdomen. Incidental findings included coronary artery disease (CAD), incomplete cor triatriatum, pericardial effusion, pleural effusion, pulmonary nodules, pulmonary infiltrates, pulmonary mass, thoracic aortic aneurysm, mediastinal nodes, abdominal mass, and liver nodules.

Conclusions: CCT is a cornerstone investigation prior to AF CA and can show multiple incidental findings, thus potentially functioning as a screening method for the detection of other significant conditions. There is still a debate whether further workup is needed or not as most findings will eventually be benign and further investigations could mean financial burden and clinical risks to the patients. Further larger prospective studies are needed with long-term follow-up to determine whether incidental findings on CCT have an impact on the long-term outcomes of patients.

## Introduction

Atrial fibrillation (AF) is the most common cardiac arrhythmia [1], affecting more than two million adults in the United States (US) [2]. AF is a major risk factor for developing ischemic stroke and accounts for 15% of all strokes in the US [3,4]. Catheter ablation (CA) has been established as a safe and effective technique for the management of drug-refractory AF [5,6]. There have been multiple clinical trials comparing AF CA with antiarrhythmic drugs including Radiofrequency Ablation vs Antiarrhythmic Drugs as First-Line Treatment of Paroxysmal Atrial Fibrillation (RAAFT-2) trial [7], Early Aggressive Invasive Intervention for Atrial Fibrillation (Early-AF) trial [8], and Catheter Ablation Versus Antiarrhythmic Drug Therapy for Atrial Fibrillation (CABANA) trial [9]. According to expert consensus, AF CA has become a class I indication for patients with symptomatic AF that is refractory or intolerant to antiarrhythmic drugs [10]. Recently, CA became an alternate initial approach in paroxysmal AF and has shown superiority over drug therapy in the prevention of atrial arrhythmia recurrence with a low incidence of serious procedural adverse events [8,11].

Cardiac computed tomography (CCT) is a rapidly evolving non-invasive imaging modality. It can provide an accurate comprehensive assessment of cardiovascular anatomy including coronary arteries. CCT has shown a great role in electrophysiological studies, especially in the use of pulmonary vein isolation for AF. CCT has been a crucial part of the planning of ablation procedures as it can allow the evaluation of left atrial and pulmonary vein anatomy and anatomy of the esophagus in order to avoid the risk of perforation during the procedure, rule out cardiac thrombus, and detection of post-procedural pulmonary vein stenosis [12-16]. CCT can frequently detect collateral findings that could be either benign or require further diagnostic investigations and even invasive procedures [17]. The aim of our study was to assess the prevalence and distribution of collateral CCT findings in patients with AF undergoing CCT prior to CA.

## Materials and methods

Data source

This is a retrospective study that reviewed 218 patients from July 2013 to June 2016 that were undergoing AF CA at Boca Raton Regional Hospital, Boca Raton, Florida, US, and Delray Medical Center, Delray Beach, Florida, US. Prior to CA, patients were referred for preprocedural CCT at the Diagnostic Centers of America, Florida, US. This study was exempted from institutional review board (IRB) approval, as it was a retrospective study that included data from medical records, and data were de-identified. 

Selection criteria

Our inclusion criteria were all patients undergoing AF CA. Exclusion criteria included patients with previous AF CA.

Data extraction and analysis

Data were extracted by one investigator (JM) who analyzed all CCT done and identified all the incidental findings.

Outcomes

Our study outcomes were the prevalence and significance of incidental CCT findings for patients undergoing AF CA.

## Results

After analyzing the CCT done over the three-year period, clinically significant incidental findings found in the chest and upper abdomen were detected in 52 out of 218 patients (23.85%). Incidental findings are shown in Table 1. Coronary artery disease was detected in 15 patients (6.88%), which would require further workup including invasive coronary angiography. There was one patient detected to have a cardiac anomaly (incomplete cor triatriatum) that has significant importance with the CA procedure. Incidental pericardial effusion was detected in two patients (0.91%) while incidental pleural effusion was detected in nine patients (4.12%). Pulmonary findings were detected in 27 patients (12.3%) including 18 patients with pulmonary nodules, eight patients (3.66%) with pulmonary infiltrates, and one patient (0.45%) with pulmonary mass. Other clinically significant findings included thoracic aortic aneurysm in three patients (1.37%), mediastinal nodes in seven patients (3.21%), abdominal mass in two patients (0.91%), and liver nodules in four patients (1.83%).

**Table 1:** Incidental CCT findings CCT: cardiac computed tomography

Organ	Findings	No. of incidental findings (%)
Cardiac	Coronary artery disease	15 (6.88%)
	Incomplete cor triatriatum	1 (0.45%)
Pericardium	Pericardial effusion	2 (0.91%)
Lung	Pulmonary nodules	18 (8.25%)
	Pulmonary infiltrates	8 (3.66%)
	Pulmonary mass	1 (0.45%)
Pleura	Pleural effusion	9 (4.12%)
Aorta	Thoracic aortic aneurysm	3 (1.37%)
Mediastinum	Mediastinal nodes	7 (3.21%)
Abdomen	Abdominal mass	2 (0.91%)
Liver	Liver nodules	4 (1.83%)
Total	All incidental findings	52 (23.85%)

## Discussion

In this retrospective observational study that included 218 patients, we analyzed CCT for patients undergoing AF CA. Our main findings showed that CCT in planning for AF CA showed incidental findings in 23.8% of patients that could need further workup.

Multiple studies have discussed the role of CCT in the detection of incidental findings of patients undergoing CA for AF. Wissner et al. studied 95 patients and found that 53% of those patients had incidental findings mostly extracardiac and more than half of these findings are pulmonary, 16% of total patients with incidental findings had to do further tests, and one patient had a newly diagnosed adenocarcinoma (1.1%) [18]. Sohns et al. evaluated 158 patients and looked only for extracardiac incidental findings and found 72% of patients had incidental findings and lung cancer was diagnosed in two patients (1.3%) [19]. Another study by Sohns et al., which involved 203 patients discovered cardiac findings in 91% of patients and extracardiac findings in 80% of patients, two newly diagnosed cancers (esophageal and pulmonary were detected; 0.9% of patients), and a newly detected aortic dissection [20]. Schietinger et al. detected extracardiac events in 69% of 149 patients, while Martins et al. detected 23% of incidental findings in 250 patients [21]. Perna et al. evaluated 173 patients; 56% of patients showed incidental findings, with newly diagnosed bronchogenic carcinoma in 1.7% of patients [17]. Compared with previous studies, our study, which included 218 patients, showed similar results but with slightly lower incidence; incidental findings were detected in 23.8% of patients mostly extracardiac, especially pulmonary lesions. This could be contributed to differences in age or the population involved.

As the number of AF CA procedures is growing, it necessities advanced cardiac imaging techniques [13]. CCT scan is an essential part of the preoperative and left atrial anatomy CA. Several studies have shown multiple incidental collateral findings with clinical significance. These findings could be cardiac or extracardiac that can include CAD, lung and esophageal cancers, pulmonary fibrosis, pulmonary emphysema, congenital cardiac abnormalities, mediastinal nodes, upper abdominal masses, or liver findings. CCT may have added utility as a screening tool for previously unrecognized yet clinically significant conditions. These findings could need additional investigations and follow-up, and could be lifesaving [13,18-23]. It is still unclear if additional cost, radiation, and invasive procedures will provide patients with mortality benefits or improved quality of life [24,25].

This study has several limitations. First, this study included a small number of patients. Second, we could not identify patients' demographics as data was de-identified. Third, it was a retrospective observational study that could only detect the prevalence of those incidental findings without long-term follow-up. Fourth, it is unclear if further workup will affect patients’ mortality and quality of life. Further larger studies are needed to better evaluate the role of CCT as a screening tool for patients undergoing AF CA.

## Conclusions

The use of CCT prior to AF CA can show multiple incidental findings that can be used as a screening method for the detection of significant conditions in the heart, lungs, and upper abdomen. These findings can necessitate further workup affecting the management and survival of these patients. Still, there is controversy about whether to manage those findings or not as the costs and risks to the patients from further investigations could exceed the benefits; especially since most of these findings will be benign. However, some of the findings could be early stages of malignant disease that if detected earlier could have a marked benefit on those patients. A prospective larger clinical study with long-term follow-up is warranted to explore the significance on patients’ outcomes.
